# Neuroretinitis as a Complication of Cat Scratch Disease

**DOI:** 10.7759/cureus.45866

**Published:** 2023-09-24

**Authors:** Jaycob Avaylon, Kimberly Lau, Kirk Harter, Azaam Mamoor, Reshma Mehendale, Leonard Ranasinghe, Edward Durant, Gurvijay Bains

**Affiliations:** 1 Clinical Sciences, California Northstate University College of Medicine, Elk Grove, USA; 2 Ophthalmology, Kaiser Permanente Central Valley, Modesto, USA; 3 Clinical Medicine, California Northstate University College of Medicine, Elk Grove, USA; 4 Emergency Medicine, Kaiser Permanente Central Valley, Modesto, USA

**Keywords:** fundoscopy, ultrasound, cat scratch disease, bartonella henselae, neuroretinitis

## Abstract

In this case report, a patient with neuroretinitis from a *Bartonella henselae* infection is described, and insights into methods to distinguish this type of case from more common etiologies of optic nerve edema are presented. A 21-year-old female with a history of right monocular vision loss due to amblyopia presented to the emergency department (ED) with occipital headache, fever, dizziness, nasal congestion, and painless blurry vision in the left eye for one day. A lumbar puncture found a slightly high opening pressure but no evidence of meningitis. The patient was diagnosed with a viral illness and discharged with outpatient follow-up. The patient, however, had persistent central vision loss and recurring headaches and returned to the ED. Subsequent ultrasound of the patient’s optic nerve revealed significant optic nerve swelling. A new working diagnosis of idiopathic intracranial hypertension was made, and the patient was started on oral acetazolamide. On the next day, she was seen by ophthalmology, and recent scratches from her cat were noted on her arm. She tested positive for *B. henselae* and was started on doxycycline and rifampin. Nearly two weeks after the initial presentation, a macular star pattern, indicative of neuroretinitis, was noted on the fundoscopic exam. The patient had recovered her vision by three months later. In ED cases with unilateral vision loss, early use of point-of-care ultrasound and infection with *B. henselae* should always be considered. Early serology testing may be warranted to allow for earlier treatment since classic signs of neuroretinitis may not be apparent at the onset.

## Introduction

Cat scratch disease (CSD) is an infectious disease typically caused by *Bartonella henselae* (*B. henselae*) contracted from a scratch or bite from an infected cat. In the United States, the incidence of CSD for people under the age of 65 is about 4.7 per 100,000 persons [1]. *B. henselae *is a gram-negative, aerobic, intracellular bacillus that is recognized as the most common causative agent of CSD, although other *Bartonella* species (*B. quintana*, *B. grahamii*, and *B. elizabethae*) are also reported to manifest CSD with ocular symptoms [2]. It has been estimated that 1-2% of cases of CSD occur with neuroretinitis. 

Neuroretinitis is classically the triad of unilateral vision loss, focal optic disc inflammation, followed by fluid spreading to the adjacent peripapillary retina, and the late formation of a macular star one to two weeks after onset [3]. Symptoms include central visual field defect on an Amsler grid testing in 88% of cases, relative afferent pupillary defect in 68% of cases, and, rarely, color vision impairment [4]. The long-term prognosis of CSD-related neuroretinitis is typically good, usually with full recovery of vision, especially for immunocompetent patients.

## Case presentation

The patient was a 21-year-old female with a history of amblyopia in the right eye and obesity who presented to the emergency department (ED) with occipital headache, fever, dizziness, nasal congestion, and painless blurry vision loss in the left eye for one day. She denied recent trauma, neck pain and stiffness, focal weakness, rash, abdominal pain, nausea or vomiting, or coughing. Her neurological exam was otherwise unremarkable, and her physical exam was similarly unremarkable, with no involuntary hip or knee flexion with passive forward flexion of the neck. As per the first physician note, the visual acuity was not done because the patient did not have her corrected lenses, but she had a baseline vision of 20/200 in her right eye, as per the patient. Computed tomography (CT) of the brain revealed no acute pathology. A lumbar puncture performed at that time showed no evidence of meningitis, but an opening pressure was not measured, due to upright positioning during the procedure and inaccuracy of an opening pressure in this position. She received supportive treatment with intravenous normal saline and oral acetaminophen for a presumed viral infection and was discharged home with plans for outpatient follow-up. 

Three days later, she returned to the ED because her central blurry vision in her left eye remained unchanged. She no longer complained of headache, fever, and dizziness. Visual acuity testing done at this time was 20/50 in the right eye, and she was unable to see at all in the left eye. Magnetic resonance imaging (MRI) and magnetic resonance venography of the head revealed no significant findings. A point-of-care ultrasound of the left eye showed an enlarged optic nerve sheath diameter (ONSD) of greater than 7 mm. A lumbar puncture was performed and showed an opening pressure of 22 cm H2O (reference < 20), normal glucose and protein levels, and no findings indicative of meningitis. Her complete blood count and serum lactate were within normal ranges. Given the abnormal findings of elevated opening pressure and vision loss, with her demographic risk factors of female sex and BMI of 42 kg/m², a neurologist was consulted and recommended treatment for idiopathic intracranial hypertension (IIH). The patient was started on oral acetazolamide 250 mg BID (two times a day), and referred to ophthalmology.

On the following day, the patient was evaluated by an ophthalmologist who noted left optic disc edema on a fundoscopic exam. The patient reported persistent central vision loss with normal peripheral vision for the previous nine days. The ophthalmologist noted that the patients visual acuity was 20/100 in the right eye and 20/200 in the left eye. The patient did have reactive pupils on examination. Following a focused history, the patient recalled a cat scratch to her right arm a few days prior to the onset of symptoms, and scar tissues from previous cat scratches were noted in the physical exam. The consulting ophthalmologist diagnosed neuroretinitis likely secondary to *Bartonella henselae*. *B. henselae* antibody titers and polymerase chain reaction were ordered, and the patient was placed on oral rifampin 300 mg twice a day and oral doxycycline 100 mg twice a day for 14 days. The doxycycline course was extended after. 

**Figure 1:**
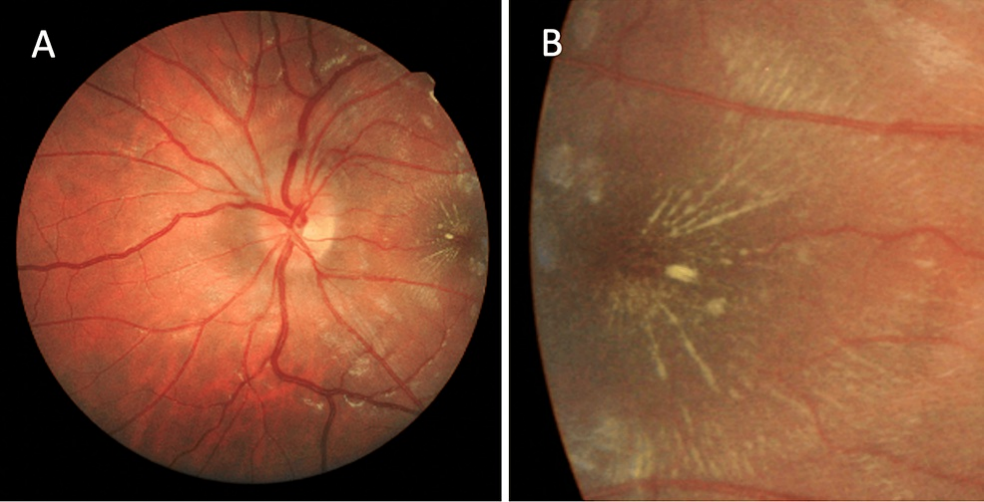
Fundus photo of the patient's left eye demonstrating optic disk edema (A) and characteristic star pattern (B), indicative of neuroretinitis.

**Figure 2:**
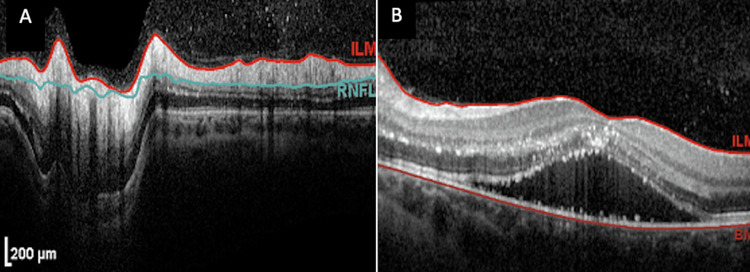
Optical coherence tomography (OCT) studies at the presentation of the patient's optic disc (A) and macula (B).

Six days later, the best corrected visual acuity (BCVA) measured was 20/200 OS. Fundoscopic exam of the left eye revealed persistent optic disc edema (Figure 1A) and exudates in a stellate pattern nasal to the macula, documented on fundoscopic photos (Figure 1B). Optical coherence tomography (OCT) around the optic disc showed edema (Figure 2A), and OCT of the macula showed subretinal fluid (Figure 2B). As per the ophthalmologist, there were no cells in the vitreous or anterior chamber. The periphery was flat and intact without any peripheral lesions. *B. henselae* serology titers were positive: IgG 1:512, IgM 1:32. The patient was advised to continue the antibiotic medications and follow-up with ophthalmology and neurology. 

**Figure 3:**
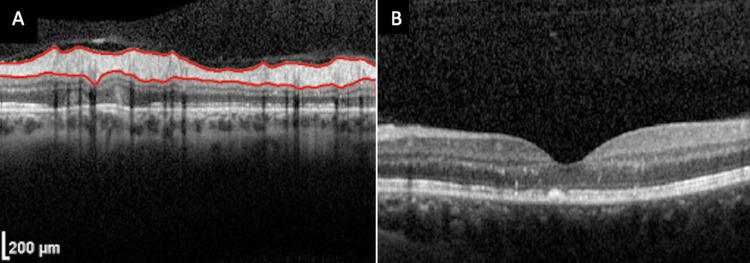
Optical coherence tomography (OTC) studies at the resolution of the patient's optic disc (A) and macula (B).

Three months after the initial presentation, the patient reported that she had improved left eye visual acuity and no new complaints. The BCVA improved to 20/20 OS. Interestingly, the amblyopia in her right eye had actually improved during this period, possibly as a result of the increased visual stimulation of the amblyopic eye, which may have acted similarly to an eye patch or visual occlusion therapy used in the treatment of amblyopia [5]. On the fundoscopic exam of the left eye, the optic disc edema had resolved, and the cup-to-disc ratio was normal at 0.2, with peripapillary atrophy. The macula was noted to have a few central exudates without elevation, which was an overall improvement over baseline fundoscopic photos. OCT of the optic disc showed normal retinal nerve fiber layer thickness, absorption of subretinal fluid, and reduced thickening of the retinal layers (Figure 3A), and OCT of the macula showed a normal foveal contour (Figure 3B).

## Discussion

The diagnosis of neuroretinitis is primarily based on fundoscopy to visualize the optic disc edema and macular star exudates [3]. OCT is used to visualize the extent of intra- and subretinal fluid accumulation. OCT imaging is especially important as it can detect macular edema before the formation of the macular star seen on fundoscopy. Laboratory testing should be ordered according to history and clinical suspicion. *B. henselae* titers, as well as fluorescent treponemal antibody absorption (FTA-ABS), rapid plasma reagin (RPR), QuantiFERON-TB Gold or purified protein derivative (PPD), chest X-ray, angiotensin converting enzyme (ACE) levels, Rocky Mountain spotted fever, and Lyme serology, can be considered, among others [6,7,8]. Further imaging, including MRI, can be considered, but it is generally not required for diagnosis. The differential diagnosis includes papilledema, hypertensive retinopathy, diabetic papillopathy, or toxic etiologies, but these usually present bilaterally [4,9].

Although point-of-care ultrasonography is commonly utilized for ocular pathologies in the ED, it is still particularly underutilized for neuroretinitis. In this case, the early utilization of ultrasound may have narrowed the differential diagnosis and led to an earlier ophthalmological referral and definitive treatment and avoided unnecessary testing, such as the second LP. Although the findings of papilledema and increased optic nerve sheath diameter on POCUS are non-specific and can be due to conditions that cause inflammation of the optic nerve and those that increase intracranial pressure, it can still be very useful. The patient presented with a unilateral visual disturbance on the first visit, which was not thoroughly investigated until four days later. The finding of widened ONSD on ultrasound, in particular, contributed to the modification of the management plan. Perhaps if ultrasound is more routinely used for ocular complaints in the ED, neuroretinitis would have been higher, and treatments could have been initiated. The patient may have been able to avoid a second LP as well. Non-ophthalmologic clinicians have reported low levels of comfort performing an accurate, nondilated fundoscopic exam [9], so the use of point-of-care ultrasound in the ED could provide a helpful alternative for the early detection of optic nerve edema. 

## Conclusions

An accurate history and physical examination, including a fundoscopic exam, is the key to differentiating between neuroretinitis and elevated intracranial pressure in patients with optic nerve edema. In this case, the patient had a history of fever, and infectious causes should have been considered in addition to IIH. Exposure to household pets was a key historical component in diagnosing neuroretinitis secondary to *B. henselae*. 

Patients with unilateral vision loss presenting to the ED may benefit from ocular point-of-care ultrasound to help identify urgent and emergent causes of vision loss, including neuroretinitis, retinal detachment, vitreous hemorrhage, and papilledema. Because most cases of infectious neuroretinitis are caused by *B. henselae*, in patients with an appropriate relevant history, early serology testing may be indicated, as classic signs of neuroretinitis.

Due to the difficulty of performing a fundoscopic exam, more modalities, such as ocular ultrasound, are valuable to aid in the early diagnosis of ocular pathology in the emergency setting.
